# Impact of Short- and Long-Term Exposure to Engineered Wood (Plywood and Particle Board) on Immune and Oxidative Biomarkers: A C57BL/6 Mouse Model Study

**DOI:** 10.3390/polym17131794

**Published:** 2025-06-27

**Authors:** Hui Ma, Keon-Ho Kim, Chang-Deuk Eom, Md. Habibur Rahman, Johny Bajgai, Sofian Abdul-Nasir, Chaodeng Mo, Won-Joong Hwang, Seong Hoon Goh, Bomi Kim, Kyu-Jae Lee, Jiyoon Yang, Cheol-Su Kim

**Affiliations:** 1 Department of Global Medical Science, Wonju College of Medicine, Yonsei University, Wonju 26426, Republic of Korea; mahui56@yonsei.ac.kr (H.M.); abdulnasirsofian62@gmail.com (S.A.-N.); chaodengmo@gmail.com (C.M.); 2 Department of Convergence Medicine, Wonju College of Medicine, Yonsei University, Wonju 26426, Republic of Korea; globaldreamer1990@gmail.com (M.H.R.); johnybajgai@yonsei.ac.kr (J.B.); forget419@hanmail.net (S.H.G.); kimbomi9090@gmail.com (B.K.); medbio@yonsei.ac.kr (K.-J.L.); 3 Department of Orthopedic Surgery, People’s Hospital of Fengjie County, Chongqing 404600, China; 4 Division of Wood Industry, Department of Forest Products and Industry, National Institute of Forest Science, Seoul 02455, Republic of Korea; keon@korea.kr (K.-H.K.); willyeom@korea.kr (C.-D.E.); wonjoung@korea.kr (W.-J.H.); 5 Department of Laboratory Medicine, Wonju College of Medicine, Yonsei University, Wonju 26426, Republic of Korea

**Keywords:** man-made wood, plywood, particle board, toxicity, inflammation, indoor air quality

## Abstract

Plywood and particle boards, commonly used in construction and interior environments, are sources of indoor chemical emissions from synthetic adhesives, resins, and surface treatments. Among these, formaldehyde, classified as a group 1 carcinogen by the International Agency for Research on Cancer, and other compounds are associated with oxidative stress, inflammation, and organ toxicity. This study aimed to evaluate the toxicological and physiological effects of plywood and particleboard emissions in female C57BL/6 mice. The mice were exposed to formaldehyde, phytoncides, and untreated wood samples under short- (30–60 days) and long-term (120–180 days) conditions. Biological effects were assessed through histopathology of major organs, differential white blood cell counts, oxidative stress markers, antioxidant enzyme activities (catalase and glutathione peroxidase), liver and kidney function tests (alanine aminotransferase, aspartate aminotransferase, blood urea nitrogen, and creatinine), and inflammatory cytokine profiling (interferon-gamma, tumor necrosis factor-α, interleukin (IL)-10, and IL-12p70). These findings revealed no significant pathological changes or systemic toxicity following long-term exposure. Minor elevations in hepatic and renal biomarkers were observed but remained within physiological limits. Antioxidant responses and cytokine fluctuations suggested mild adaptive and immunomodulatory effects. These results highlight the importance of reducing emissions from engineered wood products to improve indoor air quality and minimize potential health risks.

## 1. Introduction

Indoor air quality is increasingly recognized as a critical public health concern driven by the growing prevalence of indoor activities and the widespread use of materials that release chemical emissions [1,2]. Particleboard (PB), plywood, and medium-density fiberboard are examples of engineered wood products that frequently emit volatile organic compounds (VOCs) into their interior spaces. The leading cause of these emissions is the manufacturing process used for artificial adhesives, resins, and surface treatments. Formaldehyde is one of the most important VOCs because of its health hazards and widespread use in wood-based products [3]. The International Agency for Research on Cancer (IARC) has designated formaldehyde as a Group 1 carcinogen, or “carcinogenic to humans” [4]. The main activities that cause exposure to this chemical are the processing of chipboards or plywood panels for carpentry, building maintenance, and sanding before painting. Other workers who are most exposed are firefighters [5,6], healthcare workers [5], and beauticians.

Nonetheless, there is sufficient data from human research to support this classification, showing that formaldehyde exposure increases the risk of leukemia, particularly myeloid leukemia, and can result in nasopharyngeal cancer [6]. Additionally, formaldehyde exposure, especially by inhalation in indoor and occupational settings, poses a risk of cancer to people, according to the IARC conclusion, which was initially published in 2004 and confirmed in later evaluations [4]. The mechanisms through which formaldehyde inhalation affects allergic lung inflammation are not completely understood. In a recent study, we showed that formaldehyde inhalation increases the oxidative burst and nitration of proteins in the lungs, confirming the idea that oxidative stress plays a role in formaldehyde-induced lung inflammation [7].

These oxidants may form following the activation of nitric oxide synthases and NADPH oxidases found in structural lung and inflammatory cells [8]. Cellular defense mechanisms involving glutathione (GSH), catalase (CAT), and superoxide dismutase (SOD) control oxidative stress [9]. In the liver and kidney tissues, formaldehyde metabolites increase oxidative stress and lipid peroxidation by increasing the GSH balance [10]. Consequently, formaldehyde is frequently used to fabricate resins as binders and adhesives in engineered wood products and in other sectors, including chemical manufacturing, plastics, and textiles [6]. Consequently, it is a common indoor air pollutant, particularly in spaces containing new or recently manufactured wood-based materials [6]. Formaldehyde causes inflammation by producing reactive oxygen species (ROS) and triggering redox-sensitive pathways such as the nuclear factor kappa B (NF-κB) and mitogen-activated protein kinase. These pathways, in turn, cause the release of pro-inflammatory cytokines like interleukin 6 (IL-6) and tumor necrosis factor-alpha (TNF-α) [11].

This study aimed to investigate the toxicological and physiological effects of emissions from plywood and particleboards, focusing on immune modulation and redox balance. By exposing female C57BL/6 mice to formaldehyde, phytoncides, and untreated wood samples under both short- (30–60 days) and long-term (120–180 days) conditions, we assessed the key biological markers of toxicity, including histopathological changes, oxidative stress markers, liver and kidney function, and inflammatory cytokine profiles. These results provide critical insights into the adaptive and potential immunomodulatory effects of these indoor pollutants and emphasize the need to reduce emissions from engineered wood products to mitigate the health risks associated with indoor air pollution.

## 2. Materials and Methods

### 2.1. HS-GC/MS Analytical Conditions

VOC analysis was performed using an Agilent 7697A Headspace Sampler (Agilent Technologies, Santa Clara, CA, USA) coupled with an Agilent 8890 Gas Chromatograph (GC) (Agilent Technologies, Santa Clara, CA, USA) and an Agilent 5977C Mass Selective Detector (MSD) (Agilent Technologies, Santa Clara, CA, USA). The experimental parameters were meticulously optimized to ensure the accurate identification and quantification of VOCs emitted from the engineered wood samples.

Headspace Sampling Conditions: Headspace Sampler: Agilent 7697A Headspace Sampler (Agilent Technologies, Santa Clara, CA, USA); Incubation Temperature: 150 °C; Incubation Time: 30 min.

GC/MS Analytical Conditions: GC Column: Agilent J&W DB-624 (30 m × 0.25 mm × 1.4 µm) (Agilent Technologies, Santa Clara, CA, USA); Carrier Gas: High-purity helium at a constant flow rate of 1.5 mL/min; Ionization Method: Electron Ionization (EI) mode at 70 eV; Mass Detection Range: 29–550 *m*/*z* (Scan mode).

### 2.2. Animal Groupings

Six-week-old female mice with a mean weight of 20 ± 0.4 g were purchased from Orient Bio Inc. (Swingman, Republic of Korea). After a two-week acclimatization in a controlled environment (22 ± 2 °C, 40–60% relative humidity, 12 h light/12 h dark cycle, lights on at 07:00), mice were group-housed (*n* = 4 per cage) in standard individually ventilated polycarbonate cages with corncob bedding. All animals had ad libitum access to autoclaved tap water and a nutritionally complete laboratory rodent chow. For all groups, kiln-dried wood pieces identical to those used for exposure were cut to match the interior dimensions of each cage and positioned along the four side walls, leaving the floor and top unobstructed to avoid interference with bedding, food, or water. In total, one hundred and twenty mice were randomly divided into five groups as follows: not-treated group (NT), positive control group: phytoncide treatment group (PC), negative control group: formaldehyde treatment group (NC), experimental group 1: exposed to plywood (EG1), and experimental group 2: exposed to a PB (EG2). Each group was further subdivided into four time points based on the study duration: 30 days (*n* = 4), 60 days (*n* = 4), 120 days (*n* = 8), and 180 days (*n* = 8), resulting in 20 subgroups. All experimental procedures and protocols were approved by the Institutional Animal Care and Use Committee (IACUC) of Yonsei University, Wonju Campus, Republic of Korea (approval number: YWC-230614-1). Following treatment, the mice were euthanized, and blood was collected immediately via the retro-orbital vein into EDTA-coated tubes to prevent coagulation. Samples were centrifuged at 15,000 rpm for 5 min at 4 °C to separate the serum, which was subsequently aliquoted and stored at −80 °C for further biochemical analysis. Primary organs, including the skin, brain, liver, kidneys, and spleen, were harvested under sterile conditions. Portions of the organs were fixed in 10% neutral-buffered formalin for histological examination.

### 2.3. Phytoncide, Formaldehyde, and Wood Exposure Protocol

Plywood and PBs manufactured in Korea were selected to emulate real-world exposure scenarios associated with wood products. These materials were cut precisely to fit the dimensions of the mouse cages and affixed to the interior walls, ensuring that the top and bottom surfaces remained unobstructed to avoid direct contact with the mice. The plywood and particle board used in the test satisfied the quality performance of KS F 3101 (ordinary plywood) [12] and KS F 3104 (particle board) [13]. A 10% stock solution of each compound was diluted to a working concentration of 1 ppm for exposure to formaldehyde and phytoncide. The diluted solution (5 mL) was thoroughly mixed with autoclave-sterilized wood chips to replicate wood exposure. For the control group, the same sterilized sawdust was used, with 5 mL of sterile distilled water replacing the test solution. All other exposure conditions were identical across groups. The prepared wood chips were introduced into the cages and replaced weekly to maintain consistent exposure.

### 2.4. Measurement of Body Weight

Weekly body weight checks were conducted throughout the trial to establish a baseline. Mice were selected based on age, sex, and genetic background and divided into experimental groups for comparison. The initial body weight measurements were recorded before the experiment and served as the baseline. A standardized weighing procedure minimized stress, and weights were recorded weekly using a calibrated scale.

### 2.5. Histological Examination

Tissue samples from the skin, brain, liver, kidney, and spleen were immediately fixed in 10% formalin for at least 48 h. Following fixation, the specimens were embedded in paraffin and sectioned into 5 µm-thick slices. These sections were stained with hematoxylin and eosin (H&E) and examined using a fluorescence microscopy imaging system to evaluate histopathological changes.

### 2.6. White Blood Cell (WBC) and Its Differential Counts

Blood samples were drawn from the retro-orbital plexus and immediately transferred to EDTA-coated collection tubes. The tubes were gently rotated for 5 min using an automated roller mixer to ensure thorough mixing and prevent coagulation. Comprehensive WBC counts and differential analyses were conducted using an advanced hematology analyzer (HEMAVET HV950 FS; Drew Scientific, Erba Diagnostics, Dallas, TX, USA). This process provided a detailed quantification of lymphocytes, monocytes, neutrophils, eosinophils, and basophils, facilitating the complete evaluation of immune cell profiles.

### 2.7. Total ROS Estimation

The levels of ROS were assessed using the 2′,7′-dichlorodihydrofluorescein diacetate (DCFH-DA) assay, following the protocol provided by the manufacturer (Abcam, Cambridge, MA, USA). For this procedure, 10 μL of each sample was combined with 100 μL of a 20 μM DCFH-DA working solution in a 96-well microplate and incubated at 37 °C for 30 min. Fluorescence intensity, reflecting ROS concentrations, was then measured at excitation and emission wavelengths of 488 and 525 nm, respectively, using a DTX 880 multi-mode microplate reader (Beckman Coulter Inc., Brea, CA, USA).

### 2.8. Nitric Oxide (NO) Level Estimation

The NO concentrations were quantified using the Griess reagent assay (Promega Corp., Madison, WI, USA), according to the manufacturer’s instructions. Briefly, 50 μL of each sample was mixed with 50 μL of sulfanilamide solution in a 96-well microplate and incubated at room temperature for 10 min. Following this, 50 μL of N-(1-naphthyl) ethylenediamine dihydrochloride solution was added, and the plate was incubated for an additional 10 min at room temperature. The optical density (OD) of the resulting solution was measured at 520 nm using a SpectraMax ABS Plus microplate reader (Molecular Devices, San Jose, CA, USA).

### 2.9. Intracellular Glutathione Peroxidase (GPx) and CAT Enzyme Activity Level Estimation

The intracellular activities of the endogenous antioxidant enzymes GPx and CAT were measured using commercially available assay kits (BioVision Inc., Milpitas, CA, USA, for GPx; Biomax Co., Ltd., Guri-si, Republic of Korea, for CAT), according to the respective manufacturer’s protocols. For the CAT assay, 78 μL of the prepared sample (10 μL of stock solution and 68 μL of assay buffer) was added to each well of a 96-well microplate, while for the GPx assay, 50 μL of the prepared sample (10 μL of stock solution and 40 μL of assay buffer) was used. The plates were incubated at 37 °C for 30 min, after which optical densities were measured at 560 nm for CAT and 340 nm for GPx using a SpectraMax ABS Plus microplate reader (Molecular Devices, San Jose, CA, USA). Enzyme activity was expressed as nmol/min/mL for CAT and mU/mL for GPx.

### 2.10. Measurement of Lactate Dehydrogenase (LDH) Activity

Serum LDH activity was determined using a commercially available LDH assay kit (Biovision, Milpitas, CA, USA), following the manufacturer’s instructions. Serum samples were dispensed into a 96-well microplate, and 50 μL of reagent from the kit was added to each well. The reaction mixture was incubated at 37 °C for 30 min. Absorbance was measured at 450 nm using a SpectraMax ABS Plus microplate reader (Molecular Devices, San Jose, CA, USA). The LDH activity was quantified based on a standard curve generated using NADH concentrations ranging from 0 to 12.5 mM.

### 2.11. Alanine Aminotransferase (ALT) Activity Measurement

ALT activity was measured using a colorimetric assay kit (Biomax Co., Ltd.), according to the manufacturer’s protocol. Standard solutions were prepared with ALT concentrations of 0, 2, 4, 6, 8, and 10 nmol per well, as outlined in the instructions. For the assay, 20 μL of the serum sample was mixed with the assay buffer, standards, and 100 μL of the reaction mixture in a 96-well microplate. The plate was incubated at 37 °C for 60 min in the dark to protect the reaction from light. The OD was then measured at 570 nm using a SpectraMax ABS Plus microplate reader (Molecular Devices, San Jose, CA, USA).

### 2.12. Aspartate Aminotransferase (AST) Activity Measurement

AST activity was analyzed using a colorimetric assay kit (Biomax Co., Ltd.), following the manufacturer’s guidelines. Standard solutions were prepared at AST concentrations of 0, 2, 4, 6, 8, and 10 nM. For the assay, 50 μL of the serum sample was combined with the assay buffer, standards, and 100 μL of the reaction solution in a 96-well microplate. The OD was measured at 570 nm using a SpectraMax ABS Plus microplate reader (Molecular Devices, San Jose, CA, USA).

### 2.13. Measurement of Blood Urea Nitrogen (BUN)

BUN levels were measured using a urea nitrogen assay kit (Abbexa Ltd., Cambridge, UK), according to the manufacturer’s instructions. In the assay, 4 μL of each sample was combined with 50 μL of Enzyme Solution and mixed on an orbital shaker for 10 s. The mixture was then incubated at 37 °C for 10 min, followed by the addition of 125 µL of dye reagent. After mixing on an orbital shaker for another 10 s, the reaction was incubated again at 37 °C for 10 min. The absorbance was measured at 580 nm using a SpectraMax ABS Plus microplate reader (Molecular Devices, San Jose, CA, USA).

### 2.14. Measurement of Creatinine

Serum creatinine levels were analyzed using a creatinine assay kit (Abbexa Ltd., Cambridge, UK), according to the manufacturer’s protocol. For the assay, 12 μL of serum was combined with 180 μL of Enzyme Solution A and incubated at 37 °C for 5 min. Subsequently, 60 μL of Enzyme Solution B was added to each well, and the mixture was incubated in a 37 °C water bath. The OD values were recorded at 515 nm using a SpectraMax ABS Plus microplate reader (Molecular Devices, San Jose, CA, USA) after 2 min (OD A) and 8 min (OD B), and the results were calculated in accordance with the manufacturer’s protocol.

### 2.15. Determination of Blood Glucose and Lactate

Serum glucose and lactate levels were measured using a dedicated assay kit (Biomax Co., Ltd.), following the manufacturer’s guidelines. A 20 μL aliquot of serum was centrifuged to obtain the supernatant, which was then processed following the provided protocol. The OD was recorded at 570 nm using a SpectraMax ABS Plus microplate reader (Molecular Devices, San Jose, CA, USA).

### 2.16. IgE Level Measurement

Serum IgE levels were quantified using a human IgE ELISA kit (catalog no. ab267637), according to the manufacturer’s protocol. A 20 μL serum sample was mixed with standards, incubated at room temperature for 2.5 h, and washed three times. Subsequently, 100 μL of biotin conjugate was added, followed by 1 h of incubation and three additional washes. Streptavidin-HRP solution (100 μL) was then introduced and incubated for 45 min. Finally, 100 μL of TMB substrate was added, and the reaction was allowed to develop for 30 min. The absorbance was measured at 450 nm using a SpectraMax ABS Plus microplate reader (Molecular Devices, San Jose, CA, USA) to determine IgE concentrations.

### 2.17. Cytokine Analyses

Serum levels of the inflammatory cytokines, including interleukin (IL)-1β, IL-6, IL-10, IL-12 (p70), interferon-gamma (IFN-γ), and tumor necrosis factor-α (TNF-α), were measured using a bead-based multiplex assay kit (Bio-Rad, San Diego, CA, USA), according to the manufacturer’s protocol. Cytokine analysis was performed using a Luminex 200 Bio-Plex system, and raw data were analyzed using a five-parameter logistic regression model to ensure precise quantification.

### 2.18. Statistical Analysis Methods

Data were presented as the mean ± standard error of the mean (SEM). Statistical analyses were performed using the GraphPad Prism software (version 10.0; GraphPad, La Jolla, CA, USA). One-way analysis of variance (ANOVA) was conducted to assess differences among groups, followed by Tukey’s post-hoc multiple comparisons test, which corrects for multiple testing by controlling the family-wise error rate (FWER). All reported *p*-values were adjusted values automatically calculated by the software. Statistical significance was defined as *p* < 0.05 after adjustment.

## 3. Results

### 3.1. Characterization of VOCs Released from Plywood and Particle Board

Following the analytical report provided by the Korea Polymer Testing and Research Institute, the emissions of volatile organic compounds (VOCs) from each wood type were evaluated at four distinct time points. To standardize nomenclature, all identified VOCs were cross-checked against the PubChem “https://pubchem.ncbi.nlm.nih.gov (accessed on 31 March 2025)” database, ensuring the use of widely accepted chemical names. Additionally, their key properties, including toxicity, flammability, and potential to be explosive hazards, were assessed. A comprehensive literature review was conducted using the PubChem database to further investigate their physiological and pathological implications [14]. Based on these evaluations, VOCs were categorized into positive and negative classifications, and their relative abundance was quantified using area% for data visualization. The detailed analytical results are summarized in the Supplementary Tables S1–S4.

### 3.2. Body Weight Assessment in C57BL/6 Mice Exposed to Plywood and Particle Board Wood Sample Treatments

We continuously monitored the body weights of mice in various treatment groups throughout the study to assess the potential safety concerns associated with wood products. It was hypothesized that exposure to chemical compounds, such as formaldehyde, emitted from wood materials might influence physiological stress, metabolic processes, or disease progression, potentially resulting in observable changes in body weight under both short- and long-term exposure conditions. Despite these assumptions, the analysis revealed no statistically significant variations in body weight from the baseline to the final measurement across all groups during the 30-to-180-day observation period (Supplemental Figure S1).

### 3.3. Effects of Plywood and Particle Board Wood Samples Treatments on Major Organ Weights in C57BL/6 Mice

The purpose of this study was to investigate the physiological effects of formaldehyde emissions from plywood and PBs in C57BL/6 mice by measuring their body and major organ weights (brain, liver, spleen, and kidneys) after 30, 60, 120, and 180 days of exposure (Table 1). No significant differences in the brain or spleen weights were observed across the groups during the study period. However, the liver and kidney weights showed time-dependent variations. At 30 days, the liver weights in the NT group were significantly higher than those in the PB group, and at 60 days, they were also higher than those in the plywood group. Similarly, the kidney weights in the NT group were greater than those in the PC group at 60 days and in the plywood group at 120 days (Table 1). These findings suggest that wood exposure has a limited overall physiological impact but may lead to specific organ weight changes, warranting further investigation into the underlying mechanisms.

### 3.4. Histopathological Analysis of Skin, Brain, Liver, Kidney, and Spleen Following Plywood and Particle Board Wood Sample Treatments in C57BL/6 Mice

H&E staining was performed to evaluate the histopathological alterations in the skin, brain, liver, kidney, and spleen following the exposure to the wood products for 30–60, 120, and 180 days. Histological assessments were performed in a blind fashion by two board-certified toxicologic pathologists. Slide identities were masked using randomly generated numeric codes to prevent evaluation bias. Pathological findings were classified according to the INHAND (International Harmonization of Nomenclature and Diagnostic Criteria) guidelines; in this study, we specifically applied INHAND diagnostic criteria for the brain, liver, kidneys, spleen and skin [15,16,17,18].

Microscopic analysis of the stained sections showed no significant toxic effects or inflammatory cell infiltration in the plywood and particle board wood sample treatment groups exposed to short-term (30–60 days) or long-term (120–180 days) treatments. These findings indicated that exposure to wood products did not induce significant histopathological changes in the major organs (Supplemental Figures S2–S6).

### 3.5. Measurement of Complete Blood Cells in Blood (WBC) Following Plywood and Particle Board Wood Sample Treatments in C57BL/6 Mice

**Figure 1:**
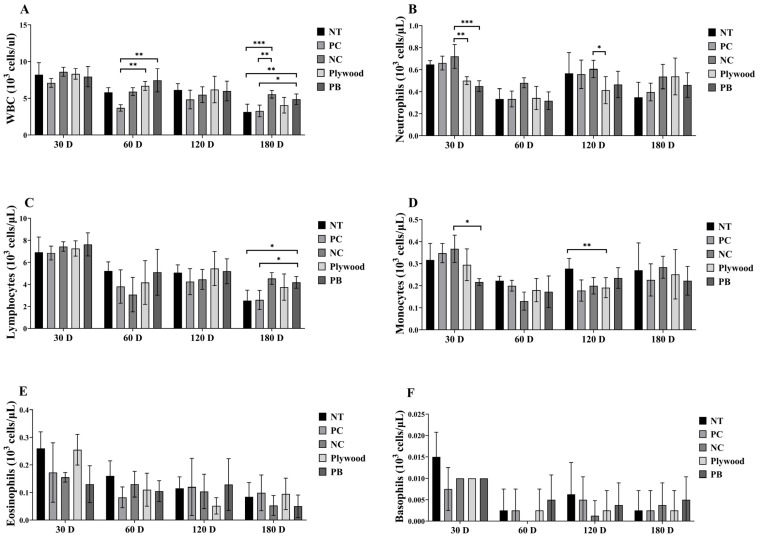
Overall data of wood product treatment for WBC and its differential counts of female C57BL/6 mice after 30, 60, 120, and 180 days of treatment. (**A**): White blood cells (WBC); (**B**): Neutrophils; (**C**): Lymphocytes; (**D**): Monocytes; (**E**): Eosinophils; (**F**): Basophils. Data were shown as mean ± SEM. * *p* < 0.05, ** *p* < 0.01 and *** *p* < 0.001 indicated statistical differences when measured with one-way ANOVA. Abbreviations: NT, untreated; PC, phytoncide-treated; NC, negative control (formaldehyde-treated); PB, particle board treated.

A temporary systemic immunological response was shown by the WBC counts, which were significantly higher in the plywood and PB exposure groups at 60 days than in the other groups but stayed mostly unchanged at 30, 120, and 180 days (Figure 1A). The NC group’s neutrophil counts increased noticeably at 30 days, but there were no discernible changes at subsequent intervals (Figure 1B). This could be an indication of a non-specific, early immune activation. The PB group did, however, have higher lymphocyte counts at 180 days, which may indicate a delayed adaptive immunological response to extended exposure (Figure 1C). Monocyte numbers differed between periods and groups. The PC group showed an increase at 30 days and variations at 120 days, indicating alterations in the dynamics of innate immune cells over time (Figure 1D).

Remarkably, the counts of neither the treatment nor control groups, eosinophil and basophil, changed significantly during the investigation, indicating that they had little role in the reaction to wood product emissions (Figure 1E,F). Together, these findings imply that immunological responses are triggered in a time-dependent and group-specific manner by exposure to particleboard and plywood emissions. Comparing WBC dynamics to the 30-day baseline, there was a trend of first fall at 60 days, rebound at 120 days, and continuing reduction at 180 days. This demonstrated how systemic immunological changes in response to extended exposure were dynamic.

### 3.6. Measurement of Oxidative Stress and Antioxidant Enzyme Activity Following Plywood and Particle Board Wood Sample Treatments in C57BL/6 Mice

**Figure 2:**
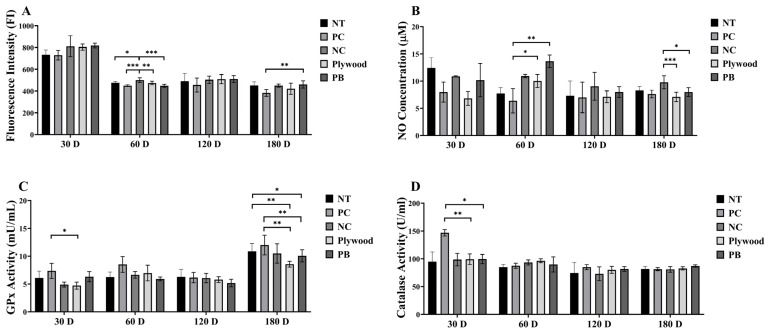
Effects of wood product treatments on oxidative stress and antioxidant enzymes markers of female C57BL/6 mice after 30, 60, 120, and 180 days of treatment. (**A**) Reactive oxygen species (ROS) concentration; (**B**) Nitrous oxide (NO) concentration; (**C**) glutathione peroxidase (GPx) activity; (**D**) Catalase activity. Data were shown as mean ± SEM. * *p* < 0.05, ** *p* < 0.01 and *** *p* < 0.001 indicated statistical differences when measured with one-way ANOVA. Abbreviations: NT, untreated; PC, phytoncide-treated; NC, negative control (formaldehyde-treated); PB, particle board treated.

ROS and NO levels, as well as GPx and catalase activity, were measured to evaluate oxidative stress (Figure 2). During the early exposure periods, ROS levels were comparatively constant; however, by 60 and 180 days, they dramatically rose in the NC and PB groups, respectively, suggesting delayed oxidative stress responses (Figure 2A). Notably, NO concentrations showed a significant increase in the plywood and PB groups at 120 days and in the NC group at 180 days, indicating a group- and time-dependent elevation (Figure 2B). These trends point to varying profiles of nitrosative stress linked to certain exposure to wood products throughout time. GPx activity was constant between groups at 60 and 120 days, but at 30 and 180 days, the plywood group’s levels were noticeably lower than those of the PC group (Figure 2C), suggesting that antioxidant defenses may be suppressed in response to specific exposures. The PC group had noticeably greater levels of CAT activity at 30 days, but there was no discernible difference in CAT activity across the groups in the latter phases (Figure 2D). Collectively, these findings highlight a time- and group-specific imbalance between oxidative stress and antioxidant defense mechanisms in response to prolonged exposure to plywood and PB emissions (Figure 2).

### 3.7. Measurement of Toxicity Markers Following Plywood and Particle Board Wood Sample Treatments in C57BL/6 Mice

**Figure 3:**
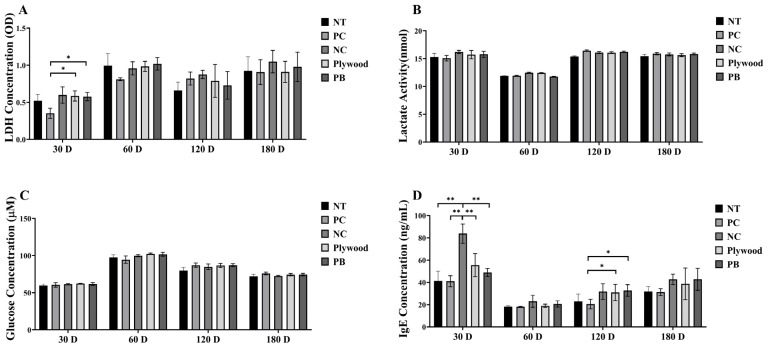
Overall data of wood product treatments on toxicity markers of female C57BL/6 mice after 30, 60, 120, and 180 days of treatment. (**A**) Lactate dehydrogenase (LDH) level; (**B**) Lactate level; (**C**) Glucose level; (**D**) Immunoglobulin E (IgE) level. Data were shown as mean ± SEM. * *p* < 0.05 and ** *p* < 0.01 indicated statistical differences when measured with one-way ANOVA. Abbreviations: NT, untreated; PC, phytoncide-treated; NC, negative control (formaldehyde-treated); PB, particle board treated.

Plywood and PB exposures were investigated for toxicity using fatigue, hepatotoxicity, nephrotoxicity, and allergy biomarkers. When compared to the control, the LDH concentrations in the plywood and PB groups were considerably higher at 30 days, indicating early cellular stress or exhaustion (Figure 3A). Figure 3B,C show that, during the course of the investigation, there were no appreciable variations in the concentrations of lactate and glucose in any of the groups tested. The plywood and PB groups showed considerably higher levels of IgE, a measure of allergic response, at 30 days, and the PB group’s levels were significantly higher than controls at 120 days (Figure 3D).

**Figure 4:**
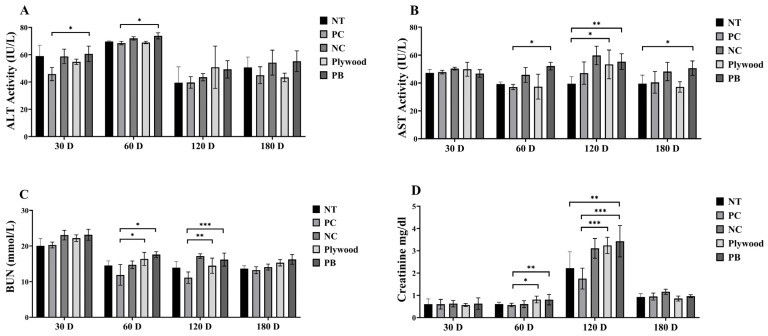
Effect of treatment of wood products on the toxicity markers of female C57BL/6 mice across 30, 60, 120, and 180 d of treatment on: (**A**): ALT level; (**B**): AST; (**C**): BUN level; (**D**): Creatinine level. Data were shown as mean ± SEM. * *p* < 0.05, ** *p* < 0.01 and *** *p* < 0.001 indicated statistical difference when measured with one-way ANOVA. Abbreviations: NT (Not treated); PC (Phytoncide-treated); NC (negative control formaldehyde-treated); PB, particle board treated.

Serum biomarkers were used to assess the hepatotoxic and nephrotoxic effects of plywood and PB exposures over several periods. At 30 and 60 days, both the plywood and PB groups showed significant increases in ALT and AST activity, which suggested early hepatocellular stress in Figure 4A,B. Interestingly, the PB group’s AST values remained noticeably higher for up to 120 days, indicating ongoing liver damage in Figure 4B. In addition, BUN and creatinine, two indicators of renal function, increased significantly after 60 and 120 days, especially in the PB group, indicating increasing nephrotoxicity Figure 4C. A declining trend was observed at 180 days, suggesting partial healing or adaptation, even though creatinine levels peaked at 120 days in Figure 4D. Throughout the trial, PB exposure generally produced more noticeable liver and renal damage than plywood.

### 3.8. Measurement of Cytokines Following Plywood and Particle Board Wood Sample Treatments in C57BL/6 Mice

**Figure 5:**
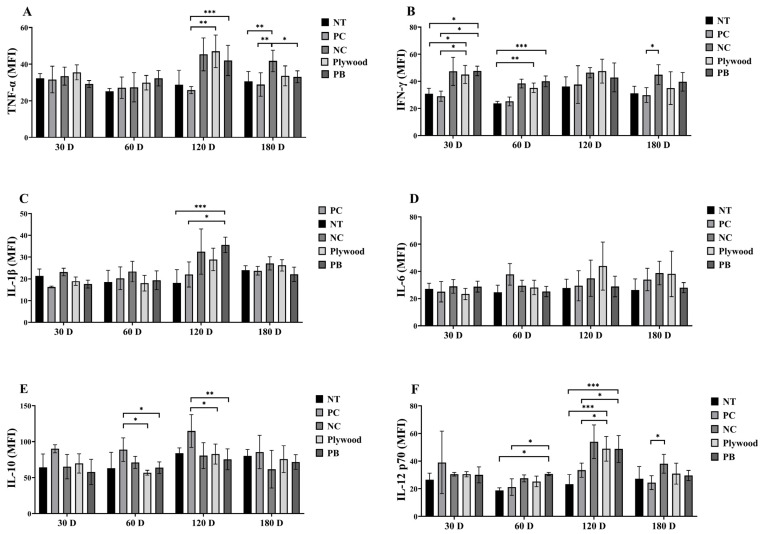
Overall data of wood product treatments for cytokines of female C57BL/6 mice after 30, 60, 120, and 180 days of treatment. (**A**): tumor necrosis factor-alpha (TNF-α); (**B**): interferon-gamma (IFN-γ); (**C**): interleukin (IL)-1β; (**D**): IL-6; (**E**): IL-10; (**F**): IL-12 (p70). Data were shown as mean ± SEM. * *p* < 0.05, ** *p* < 0.01 and *** *p* < 0.001 indicated statistical differences when measured with one-way ANOVA. Abbreviations: NT, untreated; PC, phytoncide-treated; NC, negative control (formaldehyde-treated); PB, particle board treated.

Cytokines are critical signaling molecules in the immune system that play essential roles in the regulation of immune responses, cell communication, and inflammatory processes [19]. Cytokines are essential immunological signaling molecules that coordinate inflammatory reactions and mediate cellular communication. Immune changes in the response to plywood and PB exposure were found to be material- and time-dependent based on the cytokine profile analysis (Figure 5).

At 30 and 60 days, IFN-γ levels were markedly higher in the plywood and PB groups, suggesting that Th1-mediated immune responses were activated early (Figure 5A). At 120 and 180 days, these exposed groups’ TNF-α concentrations significantly rose, indicating ongoing inflammatory activation (Figure 5B). While IL-6 levels did not significantly differ between the groups or time points (Figure 5D), the PB group’s IL-1β levels peaked at 120 days, indicating increased pro-inflammatory signaling (Figure 5C). Remarkably, at 60 and 120 days, the plywood and PB groups had considerably lower levels of the anti-inflammatory cytokine IL-10 than the PC group, suggesting that the regulatory control of inflammation was compromised (Figure 5E). Furthermore, the PB and plywood groups showed a significant increase in IL-12(p70) at 60 and 120 days, respectively, indicating a transition to a persistent pro-inflammatory state (Figure 5F). These results collectively show that wood-derived material exposure causes a dynamic imbalance in pro- and anti-inflammatory cytokines, which may have consequences for tissue stress and chronic immune activation.

## 4. Discussion

In this study, we systematically investigated the toxicological and physiological effects of plywood and PB emissions on the immune modulation and redox balance in female C57BL/6 mice following short- and long-term exposure. Our findings demonstrated that exposure to emissions from these wood products induces time-dependent alterations in systemic immunity, oxidative stress, and organ function.

While there is no direct data on the formaldehyde-induced weight changes in C57BL/6 mice, studies on other stressors, such as magnetic fields, have suggested mild weight gain in this strain [20]. In this study, mean body weights across all plywood- and particleboard–exposed groups remained within normal ranges, with only minor, non-significant fluctuations. Liver and kidney weights showed isolated increases at specific time points, but these changes lacked a consistent exposure- or dose-dependent pattern. When adjusted for body weight, relative organ weights differed by less than 10%, falling within the expected physiological range for C57BL/6 mice as reported in the baseline and toxicological reference datasets [21,22]. Crucially, no histopathological abnormalities were linked to these little deviations, which provide additional evidence that there was no chronic or subclinical organ harm at the exposure levels examined. The usefulness of the regular body and organ weight measurements as non-invasive indicators of systemic stress is demonstrated by these findings. Furthermore, the results show that the amount of VOCs released by the engineered wood products assessed in this study do not cause long-term development inhibition or compromise the integrity of visceral organs.

After extended exposure, a noticeable increase in the number of neutrophils and lymphocytes was observed, indicating a persistent inflammatory response. However, exposure to PB and plywood caused notable variations in WBC counts and their differentials. PB and plywood manufacturing workers have higher neutrophil counts than controls, most likely because of the inflammation caused by wood dust [23]. Elevated IL-1β and IL-6 stimulate the Th1/Th17 immunological pathways and neutrophil recruitment in exposed individuals [24]. Over time, formaldehyde emissions from urea formaldehyde adhesives may inhibit hematopoietic cells, lowering the number of platelets and WBCs in circulation [24]. No consistent correlation exists between the exposure duration and WBC/differential changes in tropical hardwood studies [25]. According to our findings, prolonged exposure to PB and plywood materials may have immunomodulatory effects by causing time-dependent changes in WBC profiles.

Plywood is a versatile and high value engineered wood product widely recognized as an indispensable material in construction and furniture industries. It is more environmentally friendly, cost-effective, and requires less prefabrication than other alternatives [26,27]. In contrast, PB is an engineered wood product made of wood chips joined using appropriate adhesives or synthetic resins. It is one of the most well-known wood-based goods in international trade because it is produced using a hot-pressing process with regulated pressure and temperature [28].

Formaldehyde is a well-known pro-oxidant that increases the production of ROS, compromises GPx-dependent peroxide detoxification, and speeds up lipid peroxidation by interfering with mitochondrial electron transport, impairing S-nitrosoglutathione metabolism, and lowering the GSH/GSSG ratio [29,30]. Interestingly, the most noticeable and long-lasting redox imbalance in our experiment was seen in the formaldehyde-treated cohort (NC), which was consistent with these pathways. On the other hand, mice exposed to plywood or PB emissions only showed short-term oxidative stress: GPx and CAT activity were decreased, while ROS and NO levels momentarily rose at particular periods. None of these oxidative markers, however, showed a persistent, time-dependent increase, and histological examination showed no signs of tissue damage. According to Wang et al.’s sub-chronic VOC exposure model, this redox profile shows brief oxidative shock followed by efficient physiological restoration. Before overt pulmonary or hepatic pathology developed, there were early increases in ROS and depletion of GSH [31], and the immune response was biased toward a Th2-dominant cytokine profile even when there was no discernible epithelial damage [32]. Collectively, our findings show that VOCs produced from wood are quantitatively less harmful oxidative stressors than pure formaldehyde itself. Despite temporarily undermining antioxidant defenses, they do not interfere with long-term redox equilibrium in the current exposure circumstances. Our biochemical markers’ notable agreement with earlier VOC research highlights a fundamental toxicological principle: molecular redox disruptions may occur weeks or even months before noticeable morphological alterations. Future risk assessments and regulatory research must therefore focus on determining the exposure duration, cumulative dose, and host-specific susceptibilities that convert this reversible oxidative imbalance into irreversible tissue damage.

Formaldehyde exposure increased the serum ALT and AST levels, indicating hepatocellular damage. Mechanistically, this correlates with mitochondrial dysfunction and ROS-mediated lipid peroxidation in liver tissues [33]. By activating NLRP3 inflammasomes, formaldehyde-induced ROS establishes a feedback loop between IgE-mediated inflammation and oxidative stress (ALT/AST increase) [22,23]. However, by introducing pro-inflammatory particles into deep lung tissues, wood dust from PB and plywood increases formaldehyde toxicity and exacerbates allergies and hepatic reactions [34]. At 30 days, LDH levels were considerably higher in the plywood and PB groups than in the PC group; however, no discernible changes were seen at subsequent periods (Figure 3A). There was no disturbance in energy metabolism, as shown by the constant lactate and glucose concentrations in all groups and time points (Figure 3B,C). The PB group showed a substantial rise in ALT activity at 30 and 60 days, indicating hepatocellular damage early on (Figure 3D). Hepatic stress increased with prolonged exposure, as seen by the raised AST levels in the plywood group at 120 days and in the PB group at 60, 120, and 180 days (Figure 3). These findings suggest that exposure to plywood and particleboard, particularly in the PB group, may induce transient liver stress early on, which may persist with extended exposure. Future studies will incorporate early hepatocellular stress markers, such as HO-1, NQO-1, to better characterize Nrf2-mediated antioxidant adaptation. These will be complemented by direct measures of oxidative damage (e.g., MDA, 8-oxo-dG) and new liver injury biomarkers (Glutamate dehydrogenase, High-mobility group box 1, Keratin-18) to delineate the boundary between physiological redox compensation and the onset of irreversible hepatic injury [35].

Additionally, nephrotoxic effects were shown in kidney function indicators. At 60 and 120 days, the BUN and creatinine levels in the plywood and PB groups were considerably greater than those of the PC group. Additionally, at 120 days, creatinine levels in the PB group were higher than those in the NT group (Figure 3). IgE level variations were indicative of allergic reactions. At 30 days, the plywood group’s IgE levels were noticeably higher than those of the PC group, and at 120 days, both the plywood and PB groups’ IgE concentrations were higher than those of the PC group (Figure 3). The marked elevation of IgE levels observed in this study may indicate activation of a Th2-mediated allergic response pathway. IL-4 and IL-13 are key cytokines that facilitate a class-switch recombination to IgE in B cells and promote both airway and systemic Th2-driven inflammation [36,37]. Although the expression of these Th2-related inflammatory mediators was not assessed in the current study, their evaluation represents a critical next step. Future investigations should incorporate a quantitative analysis of IL-4 and IL-13, alongside immunophenotyping of relevant Th2-associated cellular subsets—including group 2 innate lymphoid cells, peripheral Th2 cells, and IgE-producing B cells—to comprehensively elucidate the underlying immune regulatory pathways and potential sensitization mechanisms. All of these results point to the possibility of minor but escalating liver and kidney damage, as well as increased allergy reactions, from prolonged exposure to PB and plywood.

Furthermore, cytokines function in complex networks to monitor and control inflammatory and immunological responses. They are useful biomarkers for many illnesses and fall into two general categories: pro-inflammatory and anti-inflammatory cytokines [38]. IL-1β, IL-6, IL-8, IL-12, TNF-α, and interferons are examples of pro-inflammatory cytokines that increase inflammatory responses and immune effector cell activity. In contrast, anti-inflammatory cytokines that decrease inflammation and prevent immune cell activation include TGF-β, IL-4, IL-10, IL-11, and IL-13 [39]. Notably, some cytokines, such as IL-6, exhibit both pro-inflammatory and anti-inflammatory properties depending on their expression [40].

In addition, a panel of cytokines was assessed to examine their effects on inflammation and immunological modulation. At different time intervals, the plywood and PB groups showed a substantial rise in pro-inflammatory cytokines, such as IL-1β, TNF-α, IL-12, and IFN-γ, in comparison to the NT or PC groups. In contrast, the plywood and PB groups exhibited lower levels of the anti-inflammatory cytokine IL-10 than the PC group. This cytokine profile indicated a change in favor of inflammation, suggesting that exposure to VOCs from PB and plywood may cause inflammation. Pro-inflammatory cytokines have been linked to several medical disorders and are known to contribute to inflammatory processes [41]. However, low levels of the anti-inflammatory cytokine IL-10 may make it difficult for the body to control inflammation properly [42].

Our results show that C57BL/6 mice’s immune responses were dramatically changed by both brief and prolonged exposure to PB and plywood. Significantly, following 30 and 60 days of exposure, levels of pro-inflammatory cytokines—TNF-α, IL-1β, and IFN-γ—were increased, suggesting early immunological activation. At 120 days, these inflammatory alterations were still present, especially in the PB group, which had significantly greater levels of TNF-α and IL-1β than the controls. On the other hand, after 60 and 120 days, the PC group showed elevated levels of the anti-inflammatory cytokine IL-10, indicating a compensatory regulatory response. All things considered, these findings demonstrate the intricate immunological modulation, both pro- and anti-inflammatory, caused by volatile organic chemicals released by plywood and PB.

## 5. Conclusions

This study demonstrated that emissions from plywood and PB cause time-dependent immunological dysregulation, oxidative stress, and modest liver and renal damage in C57BL/6 mice. Elevated levels of pro-inflammatory cytokines, inhibited antioxidant enzymes, and elevated organ toxicity markers indicated that volatile organic chemicals, notably formaldehyde, cause systemic inflammation and redox imbalance. Increased IgE levels indicated an allergic reaction. These findings emphasize the health concerns associated with prolonged exposure to engineered wood products and the significance of emission control, safer adhesives, and routine monitoring in occupational contexts. C57BL/6 mice are useful models for testing environmental toxicity.

## Figures and Tables

**Table 1:** All data of wood product treatments for organ weight of female C57BL/6 mice.

Parameters	Groups	Treatment Time			
		30 Days	60 Days	120 Days	180 Days
Brain Weight (gram)	NT	0.463 ± 0.035	0.463 ± 0.015	0.464 ± 0.032	0.466 ± 0.028
	PC	0.453 ± 0.005	0.460 ± 0.024	0.449 ± 0.020	0.451 ± 0.028
	NC	0.473 ± 0.018	0.453 ± 0.009	0.450 ± 0.023	0.456 ± 0.038
	Plywood	0.478 ± 0.025	0.455 ± 0.005	0.459 ± 0.029	0.450 ± 0.010
	PB	0.453 ± 0.005	0.450 ± 0.014	0.476 ± 0.029	0.448 ± 0.021
Liver Weight (gram)	NT	0.883 ± 0.161	1.19 ± 0.221	1.33 ± 0.229	1.30 ± 0.182
	PC	1.13 ± 0.152	1.13 ± 0.238	1.26 ± 0.183	1.26 ± 0.134
	NC	1.05 ± 0.101	1.21 ± 0.075	1.35 ± 0.175	1.34 ± 0.203
	Plywood	0.975 ± 0.023	1.29 ± 0.104 *	1.07 ± 0.147	1.34 ± 0.146
	PB	1.16 ± 0.115 *	1.28 ± 0.225	1.19 ± 0.081	1.19 ± 0.160
Kidney Weight (gram)	NT	0.30 ± 0.047	0.340 ± 0.008	0.364 ± 0.029	0.333 ± 0.048
	PC	0.280 ± 0.024	0.275 ± 0.020 *	0.309 ± 0.050	0.333 ± 0.032
	NC	0.290 ± 0.034	0.288 ± 0.036	0.319 ± 0.035	0.330 ± 0.025
	Plywood	0.275 ± 0.050	0.308 ± 0.033	0.281 ± 0.024 ***	0.335 ± 0.028
	PB	0.283 ± 0.022	0.313 ± 0.033	0.311 ± 0.025	0.319 ± 0.027
Spleen Weight (gram)	NT	0.082 ± 0.0206	0.082 ± 0.0222	0.103 ± 0.0128	0.118 ± 0.0423
	PC	0.082 ± 0.0126	0.082 ± 0.0222	0.113 ± 0.0306	0.105 ± 0.0273
	NC	0.080 ± 0.0216	0.825 ± 0.0222	0.101 ± 0.0236	0.104 ± 0.0207
	Plywood	0.095 ± 0.019	0.113 ± 0.0095	0.088 ± 0.0173	0.103 ± 0.0238
	PB	0.0875 ± 0.0150	0.100 ± 0.0294	0.093 ± 0.0130	0.093 ± 0.0272

Data were presented as mean ± SEM. Statistical significance was determined using one-way ANOVA. * *p* < 0.05 and *** *p* < 0.001 indicate significant differences compared with the NT group determined by one-way ANOVA. Abbreviations: NT, untreated; PC, phytoncide-treated; NC, negative control (formaldehyde-treated); PB, particle board treated.

## Data Availability

All data analyzed in this study are included in this article.

## Supplementary Materials

The following supporting information can be downloaded at: https://www.mdpi.com/article/10.3390/polym17131794/s1, Figure S1. Short-term (30–60 days) and Long-term (120–180 days) effects of VOCs released from wood samples in the mice cages on body weight. Figures S2–S6. The H&E staining of the skin, brain, liver, kidney, and spleen. Tables S1–S4. GC-MS data analysis of plywood and PB.
