# Supplementary material for: Impact of Short- and Long-Term Exposure to Engineered Wood (Plywood and Particle Board) on Immune and Oxidative Biomarkers: A C57BL/6 Mouse Model Study
Source: Polymers (Basel). 2025 Jun 27;17(13):1794. doi: 10.3390/polym17131794 (PMC12252340; doi:10.3390/polym17131794)
Supplement: Supplementary file 1 [file polymers-17-01794-s001.zip › polymers-3711697-supplementary.pdf]

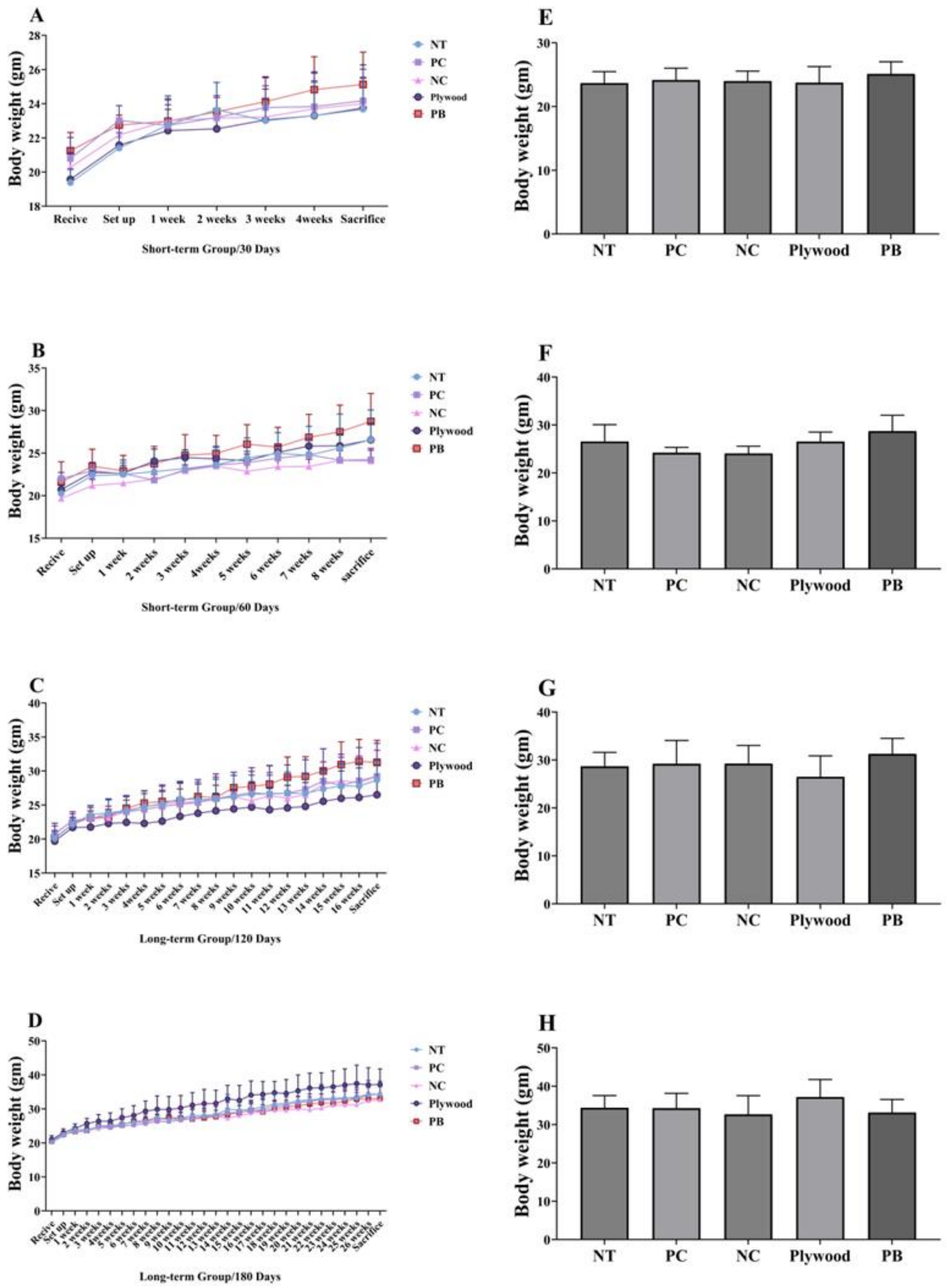

Figure S1. Short-term 30-60 days and Long-term 120-180days effects on body weight of VOCs released from wood samples in the mice cages. A-D: the weekly body weight measurement data of 30-180days; E-H: Body weight measurement data of 30-180days before sacrifice. Data is shown as mean ± SD for n=4 mice for short-term groups and n=8 mice for long-term groups.

Abbreviations: NT (Not treat); PC(Phytoncide); NC(Formaldehyde); PB (Particle Board) treated group.

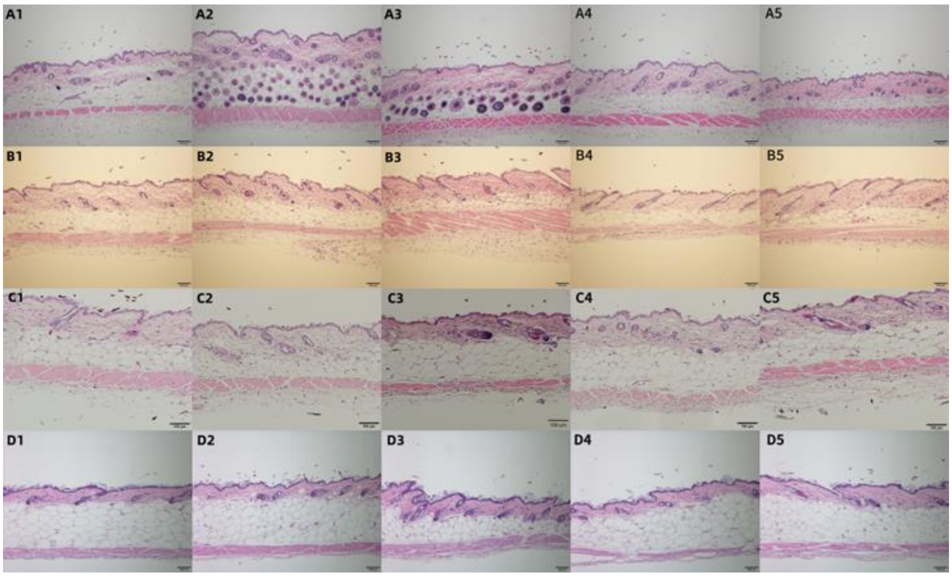

Figure S2. The H&E Staining of Skin. A: 30days; B: 60days; C: 120days; D:180days; 1: NT group; 2: PC group; 3: NC group; 4: Plywood group; 5: PB group; Hematoxylin and eosin (H&E) staining, bar=50μm; bar=100μm; bar=200μm.

Abbreviations: NT (Not treat); PC(Phytoncide); NC(Formaldehyde); PB (Particle Board) treated group.

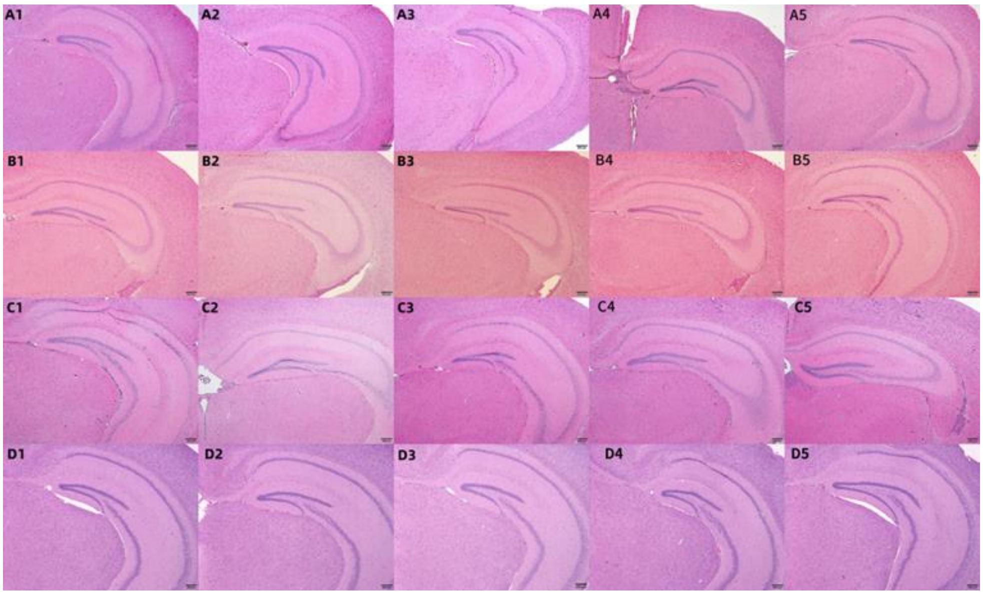

Figure S3. The H&E Staining of Brian. A: 30days; B: 60days; C: 120days; D:180days; 1: NT group; 2: PC group; 3: NC group; 4: Plywood group; 5: PB group; Hematoxylin and eosin (H&E) staining, bar=50μm; bar=100μm; bar=200μm.

Abbreviations: NT (Not treat); PC(Phytoncide); NC(Formaldehyde); PB (Particle Board) treated group.

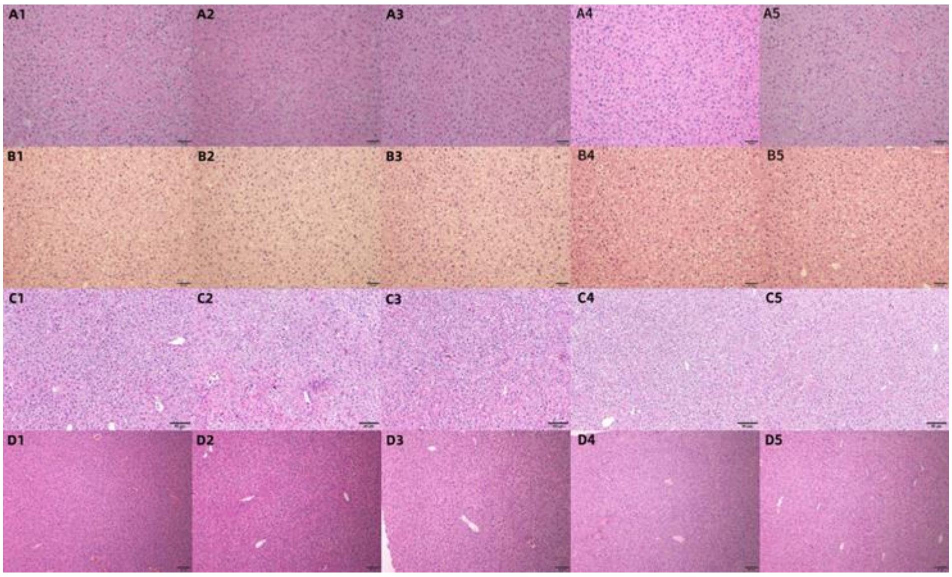

Figure S4. The H&E Staining of Liver. A: 30days; B: 60days; C: 120days; D:180days; 1: NT group; 2: PC group; 3: NC group; 4: Plywood group; 5: PB group; Hematoxylin and eosin (H&E) staining, bar=50μm; bar=100μm; bar=200μm.

Abbreviations: NT (Not treat); PC(Phytoncide); NC(Formaldehyde); PB (Particle Board) treated group.

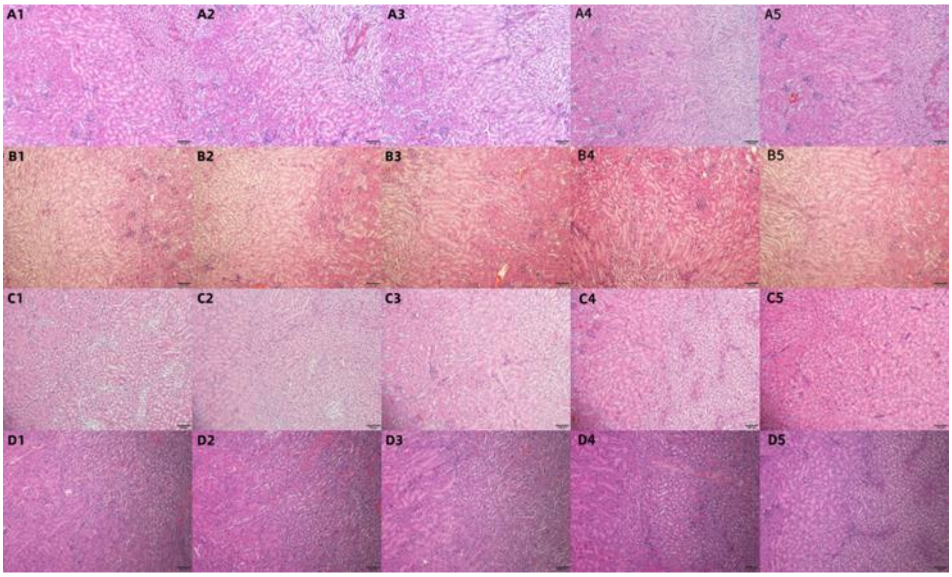

Figure S5. The H&E Staining of Kidney. A: 30days; B: 60days; C: 120days; D:180days; 1: NT group; 2: PC group; 3: NC group; 4: Plywood group; 5: PB group; Hematoxylin and eosin (H&E) staining, bar=50μm; bar=100μm; bar=200μm.

Abbreviations: NT (Not treat); PC(Phytoncide); NC(Formaldehyde); PB (Particle Board) treated group.

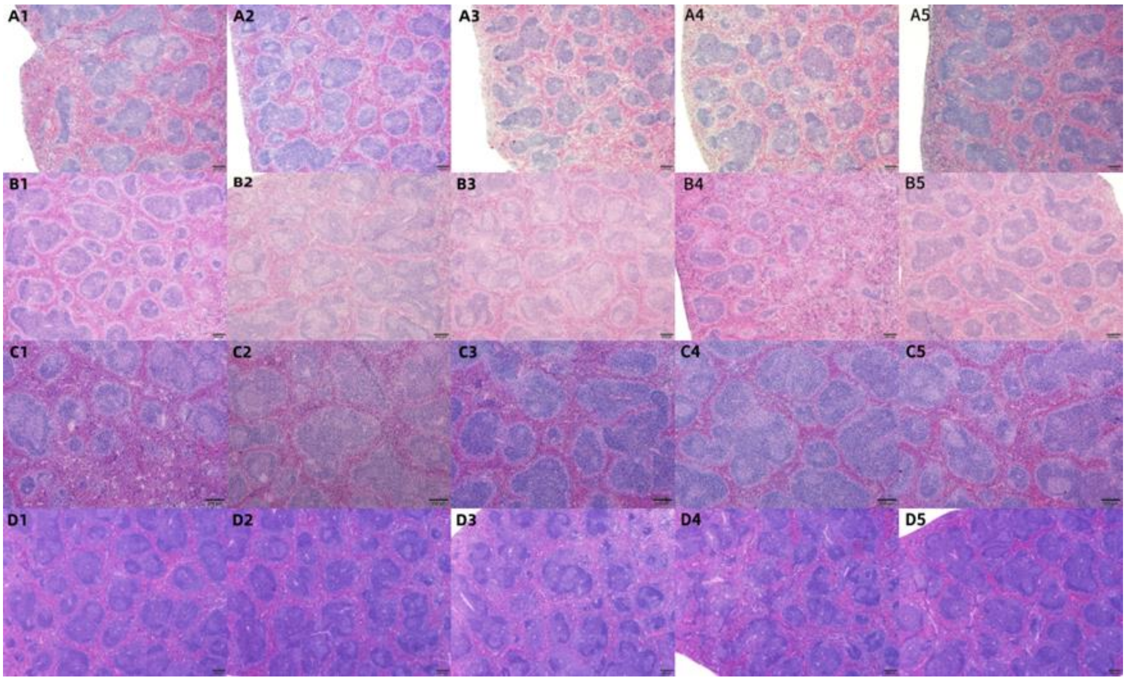

Figure S6. The H&E Staining of Spleen. A: 30days; B: 60days; C: 120days; D:180days; 1: NT group; 2: PC group; 3: NC group; 4: Plywood group; 5: PB group; Hematoxylin and eosin (H&E) staining, bar=50μm; bar=100μm; bar=200μm.

Abbreviations: NT (Not treat); PC(Phytoncide); NC(Formaldehyde); PB (Particle Board) treated group.

Table S1. Trend analysis table for positive VOCs for Plywood.

Compound	30 Days (%)	60 Days (%)	120 Days (%)	180 Days (%)
beta-pinene	20.5	26.5	21.01	29
alpha-Pinene	19.5	20.7	17.92	21.4
Acetic acid	10.6	11.5	8.66	9.3
alpha-Terpineol	4.4	4.9	3.2	2.5
D-Limonene	3.3	3.1	2.28	2.2
Benzaldehyde	2.2	-	1.10	1.5
Pinocarveol	2.3	-	2.82	-
(1R)-(-)-Myrtenal	1.8	-	-	-
Methenamine	1.7	0.9	0.59	0.8
Camphene	1.7	-	-	1.1
Fenchyl acetate	1.1	0.8	-	-

(+)-gamma-cadinene	1	-	-	-
alpha-Cadinol	0.9	-	0.98	-
Pinocarvone	0.8	0.4	1.42	-
Terpinen-4-ol	0.6	-	-	1.1
L-alpha-Terpineol	-	-	3.2	-
L-trans-Pinocarveol	-	-	2.82	-
beta-Calacorene	-	-	0.55	-
L-trans-Pinocarveol	-	-	2.82	-
m-Cymene	-	-	-	0.8
o-Cymene	-	1	0.98	-
beta-Cadinene	-	0.8	-	-
tau-Muurolol	-	0.6	-	-
endo-Borneol	-	0.4	-	-
beta-Pinene	0.6	-	2.96	-
alpha-Terpilene	0.4	-	-	-
alpha-Phellandrene	-	-	0.72	-
Sabinene	1.4	1.1	2.53	-
Fenchol	0.8	-	0.43	-
Thymol methyl ether	0.7	-	-	-
alpha-Longipinene	1.3	1.3	1.65	1.3
(+)-Cyclosativene	-	0.8	-	-

Table S2. Trend analysis table for negative VOCs for Plywood.

Compound	30 Days (%)	60 Days (%)	120 Days (%)	180 Days (%)
Methanol	7.9	8	5.48	6.5
Acetone	3.3	1.7	3.03	2.2
Furfural	1.5	1.2	1.2	-
Hexanal	1	0.9	2.56	-
1H-Pyrrole-2-carboxaldehyde	0.4	0.3	-	-

Styrene	-	0.5	-	-
Ethanol	-	-	1.77	1.6
Hexanoic acid	-	-	0.71	0.7
3,5-Dimethylpyrazole	-	-	1.1	

Table S3. Trend analysis table for positive VOCs for Particle board

Compound	30 Days (%)	60 Days (%)	120 Days (%)	180 Days (%)
Methenamine	23.2	17.5	18.5	12
Acetic acid	17.6	14.8	14.26	10.1
alpha-Pinene	7	20.8	13.4	23.8
Benzaldehyde	5.1	-	1.1	-
beta-Pinene	-	10	-	15.5
Longifolene	-	-	5.4	-
alpha-Terpineol	-	-	-	-
D-Limonene	-	-	-	-
Pinocarveol	-	-	2.82	-

Table S4. Trend analysis table for negative VOCs for Particle board

**Compound**	**30 Days (%)**	**60 Days (%)**	**120 Days (%)**	**180 Days (%)**
Methanol	8.2	8.1	6.76	5.4
Furfural	3.5	2.5	-	-
Acetone	3.4	2.5	5.26	4.7
Dimethyl phthalate	-	2.5	-	-
Styrene	-	0.8	-	-
Ethanol	-	-	2.06	1.9
Hexanal	-	-	0.41	0.3
Chloromethane	-	-	-	0.9
Propane	-	-	-	0.6

o-Isopropenyltoluene	-	-	-	0.5
Toluene	-	-	-	0.4
Hexanoic acid	-	-	-	0.3
